# New Antibiotics for *Staphylococcus aureus* Infection: An Update from the World Association of Infectious Diseases and Immunological Disorders (WAidid) and the Italian Society of Anti-Infective Therapy (SITA)

**DOI:** 10.3390/antibiotics12040742

**Published:** 2023-04-12

**Authors:** Susanna Esposito, Francesco Blasi, Nigel Curtis, Sheldon Kaplan, Tiziana Lazzarotto, Marianna Meschiari, Cristina Mussini, Maddalena Peghin, Carlos Rodrigo, Antonio Vena, Nicola Principi, Matteo Bassetti

**Affiliations:** 1 Pediatric Clinic, Pietro Barilla Children’s Hospital, Department of Medicine and Surgery, University Hospital of Parma, 43126 Parma, Italy; 2 Department of Pathophysiology and Transplantation, Università degli Studi di Milano, 20122 Milan, Italy; 3 Respiratory Unit and Cystic Fibrosis Center, Fondazione IRCCS Ca’ Granda Ospedale Maggiore Policlinico Milano, 20122 Milan, Italy; 4 Department of Paediatrics, The University of Melbourne, Parkville, VIC 3010, Australia; 5 Department of Infectious Diseases, The Royal Children’s Hospital Melbourne, Parkville, VIC 3010, Australia; 6 Division of Infectious Diseases, Department of Pediatrics, Baylor College of Medicine, Houston, TX 77030, USA; 7 Division of Microbiology, IRCCS Azienda Ospedaliero-Universitaria di Bologna, 40138 Bologna, Italy; 8 Infectious Diseases Unit, Azienda Ospedaliero-Universitaria of Modena, 41124 Modena, Italy; 9 Infectious and Tropical Diseases Unit, Department of Medicine and Surgery, University of Insubria-ASST-Sette Laghi, 21110 Varese, Italy; 10 Department of Pediatrics, Hospital Universitari Germans Trias i Pujol, Carretera de Canyet, 08916 Barcelona, Spain; 11 Germans Trias i Pujol Research Institute, Carretera de Can Ruti, Camí de les Escoles, 08916 Badalona, Spain; 12 Division of Infectious Diseases, Department of Health Sciences (DISSAL), University of Genova, 16132 Genoa, Italy; 13 IRCCS Ospedale Policlinico San Martino, 16132 Genoa, Italy; 14 Università degli Studi di Milano, 20122 Milan, Italy

**Keywords:** antibiotics, anti-infective therapy, antimicrobial resistance, MRSA, MSSA, *Staphylococcus aureus*

## Abstract

*Staphylococcus aureus* is an extremely virulent pathogen that is capable of quickly evolving and developing antibiotic resistance. To overcome this problem, new antibiotics have been developed. Some of these have been licenced for use in clinical practice, mainly for the treatment of adults with acute skin and soft tissue infections, in addition to both community-acquired pneumonia (CAP) and nosocomial pneumonia (hospital-acquired bacterial pneumonia and ventilator-associated bacterial pneumonia). In this paper, the main characteristics and clinical use of new licenced anti-staphylococcal drugs have been discussed. In vitro studies have demonstrated that some new anti-staphylococcal antibiotics have better antimicrobial activity and, at least in certain cases, more favourable pharmacokinetic properties and higher safety and tolerability than the presently available anti-staphylococcal drugs. This suggests that they may have a potential use in reducing the risk of failure of *S. aureus* therapy. However, an in-depth analysis of microbiological and clinical studies carried out with these new drugs seems to indicate that further studies need to be conducted before the problem of resistance of *S. aureus* to the antibiotics available today can be completely solved. Considering the overall available research, the drugs that are active against *S. aureus* appear to present a great therapeutic opportunity for overcoming resistance to traditional therapy. There are advantages in the pharmacokinetic characteristics of some of these drugs and they have the potential to reduce hospital stays and economic costs associated with their use.

## 1. Introduction

*Staphylococcus aureus* is a versatile pathogen. It is both a commensal bacterium and a human pathogen capable of causing a wide range of diseases. Up to 40% of the human healthy population carries *S. aureus*, with the nose, throat, skin, and intestinal tract being the most common sites of detection [1,2]. The prevalence of *S. aureus* carriage is higher in: children and older people; immunocompromised subjects, including those with allelic variants of some genes that code for factors of innate immunity; patients with chronic severe underlying disease, such as diabetes, hepatitis, and HIV; and people living in industrialized countries [3,4]. Although carriage is generally asymptomatic, under certain conditions, *S. aureus* can cause a wide range of nosocomial and community-acquired diseases. These can vary from minor skin infections, such as pimples, impetigo, boils, cellulitis, scalded skin syndrome, folliculitis, and abscesses, to life-threatening conditions, such as pneumonia, meningitis, osteomyelitis, endocarditis, toxic shock syndrome, and bacteraemia [5]. Most infections occur in carriers. However, since this pathogen is readily transmitted from carriers to other individuals, it is relatively common that infection can develop in individuals who were previously noncarriers, particularly in the healthcare environment [6]. In addition to being extremely virulent, *S. aureus* has proven to be capable of quickly evolving and developing resistance to nearly all antibiotics used to kill it. Resistance to penicillin, the first antibiotic determined to be effective against *S. aureus*, was reported only one year after the introduction of the drug in clinical practice [7]. Moreover, approximately 10 years later, it was shown that 50% or more of *S. aureus* strains detected in large hospitals were able to produce penicillinase and were penicillin-resistant, limiting the use of this drug only to the few cases in which the pathogen remained susceptible [8,9]. Equally rapid was the development of resistance to other antibiotics that were progressively entering clinical use, such as erythromycin, streptomycin, and tetracyclines [10,11]. The development of semisynthetic penicillins, such as methicillin, oxacillin, cloxacillin, dicloxacillin, and nafcillin, stable to hydrolysis by the *S. aureus* penicillinase enzyme [12], seemed to solve the problem. This is because most *S. aureus* strains, generally defined as methicillin-susceptible *S. aureus* (MSSA) strains, remained susceptible to these drugs for some decades. The detection of methicillin-resistant *S. aureus* (MRSA) increased slowly and was almost exclusively evidenced in hospitals, thus making it relatively easy to identify individuals at risk of therapeutic problems [13]. Unfortunately, starting from the end of the last century, MRSA isolates were detected, even in the community, in patients who had no recent contact with an environment where MRSA infection was expected. It was reported that the prevalence of MRSA in hospitals between 1997 and 1999 was 22.4% in Australia, 66.8% in Japan, 34.9% in Latin America, 40.4% in South America, 32.4% in the USA, and 26.3% in Europe; however, there were significant differences between countries. [14,15,16]. To overcome this problem, new antibiotics have been developed against MRSA. Among them, the most widely used are vancomycin (VAN), daptomycin, and linezolid. However, despite the recommendation to prescribe these drugs only in selected individuals to reduce the risk of resistance developing, new problems related to the treatment of *S. aureus* infection have emerged. Increased minimal inhibitory concentrations (MICs) of VAN have been reported, with strains showing levels considered to be of intermediate resistance or fully resistant because of the presence of several genetic mutations [17]. Strains with an MIC ≤ 2 mg/L are considered susceptible, those with an MIC of 4–8 mg/L are considered to be of intermediate susceptibility, and those with an MIC ≥16 mg/L are considered resistant. Risk of treatment failure has already been evidenced by the presence of strains showing intermediate resistance. On the other hand, increasing the VAN dosage to achieve higher concentration levels has not been possible due to the risk of the development of very severe adverse events. [18,19]

A reduction in activity has also been reported for linezolid, with even point mutations leading to resistance [20]. Finally, cases of daptomycin-resistant *S. aureus* have been reported [21].

Several new antibiotics have been developed to overcome the present limitations in treating *S. aureus* infection. Some of these have been licensed for use in clinical practice, mainly for the treatment of adults with acute skin and soft tissue infections (aSSTIs), in addition to both community-acquired pneumonia (CAP) and nosocomial pneumonia (hospital-acquired bacterial pneumonia [HABP] and ventilator-associated bacterial pneumonia [VABP]). Recently published reviews have summarized the microbiological and clinical characteristics of some of these new drugs [22,23,24,25]. However, only one of these reviews was specifically devoted to *S. aureus* and none of them reported a detailed analysis of the most recent studies concerning the efficacy, safety, and tolerability of all of the licensed preparations. As knowledge of these characteristics seems essential for a proper use of these new drugs, this paper has been prepared. It discusses what is presently known about the anti-*S. aureus* drugs that are already authorized for use in humans by the FDA and/or EMA. A group of experts from the World Association of Infectious Diseases and Immunological Disorders (WAidid) and the Italian Society of Anti-Infective Therapy (SITA) selected and analysed all of the studies listed by PubMed over the past 15 years, identifying those antibiotics with predominant activity against Gram-positive cocci. Among them, the clinical or, if not available, in vitro studies that were published on the use of these antibiotics for the indications mentioned above were evaluated. In addition, clinical trials conducted among paediatric patients (<18 years) were reviewed.

## 2. Cephalosporins

First-generation cephalosporins, such as cefazolin, have been largely used in the past to treat *S. aureus* infections [26]. Unfortunately, the emergence of MRSA has excluded their use for a high percentage of *S. aureus* infections. This is particularly true in severe cases for which *S. aureus* is a likely pathogen but for which no organism has been isolated and thus antibiotic susceptibilities could not be evaluated or molecular tests could not identify MSSA or MRSA as the aetiology. However, two recently developed drugs in this group, ceftobiprole and ceftaroline, have characteristics that seem to overcome the limits of the older parent molecules.

### 2.1. Ceftobiprole

Ceftobiprole (CEF) is a fifth-generation cephalosporin with a wide spectrum of antimicrobial activity, including against Gram-positive and Gram-negative bacteria. A prominent characteristic of this drug is its activity against MRSA. This is because CEF can inhibit a number of penicillin-binding proteins (PBPs) that are resistant or poorly sensitive to conventional beta-lactams, including PBP2a of MRSA. In vitro studies have shown that 99.2–100% of MRSA strains are susceptible to CEF with an MIC 50 and MIC 90 of 0.5 mg/L and 1–2 mg/L, respectively. MRSA strains with MICs of 4 mg/L or greater are considered ceftobiprole-resistant [27,28,29,30]. Similar to other beta-lactams, CEF exhibits time-dependent antibacterial activity. It is poorly bound to plasma proteins (16%), has a short elimination half-life of approximately 3 h, and is required to be administered intravenously (IV).

Based on the pharmacokinetic and pharmacodynamic characteristics of CEF, effective serum and tissue concentrations at 30–60% of the dosing interval can be achieved in healthy adults infusing 500 mg over 2 h q8h [31]. Dosage adjustments are required in patients with renal insufficiency [27]. Despite it not being approved for use in the USA [32] CEF is approved by the EMA for use in Europe against CAP, HABP, and aSSTIs in adults [33]. Two randomized controlled trials (RCTs) have tested the efficacy of CEF for the treatment of CAP and HABP [34,35]. In the first [30], adult patients with CAP requiring hospitalization were enrolled and randomly assigned to receive CEF or ceftriaxone with or without linezolid. Clinical and microbiological noninferiority of CEF compared with ceftriaxone ± linezolid was demonstrated. Among the 469 clinically evaluable patients, cure rates were 86.6% vs. 87.4%, respectively (95% confidence interval [CI] of the difference, −6.9% to 5.3%). Microbiological eradication rates were shown in 88.2% of the patients treated with CEF and in 90.8% of those receiving comparator drugs (95% CI of the difference, −12.6% to 7.5%). However, no definitive conclusion on the efficacy of CEF in *S. aureus* cases could be drawn, as the total number of cases of CAP due to this pathogen was extremely low (only 12 MSSA and 1 MRSA). Safety and tolerability were generally good for both treatments. The discontinuation of therapy was necessary in 6% of CEF patients and in 4% of subjects enrolled in the comparator group (95% CI of the difference, −1.2% to 5.4%). Treatment-associated adverse events were slightly higher in the CEF group than in the comparator group (36% vs. 26%; 95% CI of the difference, 2.9% to 17.2%), mainly due to the higher frequency of nausea and vomiting. In the second study [31], adult patients with HABP and VABP were enrolled and treated with CEF or ceftazidime plus linezolid. Cure was achieved in a similar number of clinically evaluable patients with HABP (69.3% vs. 71.3%; 95% CI, −10.0 to 6.1), showing noninferiority of CEF compared with the combined antibiotic treatment. Microbiological eradication rates in these patients were also similar, including cases due to *S. aureus*, and both MSSA and MRSA (62.9% vs. 67.5%, 95% CI, −16.7 to 7.6). In contrast, the noninferiority of CEF was not demonstrated in VABP patients. However, cure and mortality rates in mechanically ventilated patients who did not have VABP were in favour of CEF or were comparable to those for ceftazidime plus linezolid. This led the authors to conclude that factors other than the different antimicrobial efficacies were the main causes of the results shown in VABP patients. On the other hand, a pharmacokinetic analysis concluded that CEF plasma concentrations of VABP patients were sufficient to eliminate pathogens with an MIC of 4 mg/L in 92% of patients, clearly highlighting the potential efficacy of this drug.

Two randomized, double-blind studies by the Noel group have contributed to the evaluation of CEF for the treatment of aSSTIs [36,37]. In the first study [32], CEF was compared to vancomycin, and in the second study [37], CEF was compared with the combination of vancomycin plus ceftazidime. In both studies, CEF met the predetermined criteria for noninferiority in all populations analysed. The results of the first study showed a cure rate 7–14 days after the end of therapy of 93.3% among the CEF patients and 93.5% among those given comparators [36]. The infecting pathogen was eradicated in 77.8% and 77.5% of the patients, respectively. When MRSA was detected, eradication occurred in 91.8% of patients receiving CEF and 90.0% of those receiving vancomycin plus ceftazidime. Very similar results were reported in the second study [37]. Cure rates were approximately 90% with both treatments, regardless of the type of aSSTI. In *S. aureus* cases, cure rates were 92.3% for CEF and 91.4% for the vancomycin plus ceftazidime combination. In cases due to MRSA, the cure rate was only slightly lower, at 89.7% and 86.1%, respectively. The rates of adverse events and serious adverse events in the two treatment groups were similar, with incidences not dissimilar from those reported in the CAP study [34]. The pharmacokinetics and safety of ceftobiprole have been studied in children aged 3 months to 17 years old with pneumonia, but it is not approved for use in patients <18 years old.

### 2.2. Ceftaroline

Ceftaroline (CET) is an intravenous, bactericidal cephalosporin that is licenced by the EMA for the treatment of adults and children (including newborn babies) with complicated SSTIs and CAP (among *S. aureus* cases, only MSSA strains are included) [38]. In the USA, this drug has slightly different indications; it is licenced in cases of aSSTIs for adults and for children with a gestational age of ≥34 weeks and a postnatal age of ≥12 days, and in cases of CAP for adults and children aged ≥2 months old [39]. As the drug is poorly soluble, it is administered as a prodrug, ceftaroline fosamil, which is rapidly hydrolysed by plasma phosphatases to its active form [40]. This active form has a mechanism of action exactly the same as CEF, i.e., with a very high binding affinity to PBP-2a. CET is marginally bound to plasma protein and is primarily eliminated by the kidney, which explains why dosage adjustments are needed in patients with reduced renal function. CET is a time-dependent antibiotic effective against several multi-resistant Gram-positive and Gram-negative strains, including MRSA. For this pathogen, initial evaluations have shown that the MIC 50 and MIC 90 were 0.5 mg/L and 1 mg/L, respectively, values that were 1–2 dilutions higher than the MICs for MSSA [41,42].

Taking into account the pharmacokinetic and pharmacodynamic characteristics of the drug, a dosage of 600 mg every 12 h infused within 1 h, for 7–14 days for aSSTIs and for 5–7 days for CAP, has been suggested for adults with normal renal function. The potential efficacy of regimens was demonstrated by the evidence that CEF was found to be noninferior to vancomycin plus aztreonam in the treatment of various aSSTIs and to ceftriaxone for the treatment of CAP [43,44]. Regarding aSSTIs, clinical cure rates in the pooled microbiologically evaluable populations enrolled in two randomized, double-blind, multicentre trials were similar in the CET and comparator groups, including in the cases due to MSSA (93.0 vs. 94.5%) and MRSA (93.4% vs. 94.3%). For CAP, the pooled cure rates shown in two randomized, double-blind, multicentre trials were 84.3% vs. 77.7% (95% CI of the difference 1.6–11.8%) in the CET and comparator groups, respectively. For patients with *S. aureus*, clinical cure occurred in 72.0% of CET patients and in 55.6% of those given ceftriaxone. However, there was no differentiation of MSSA from MRSA [45]. However, when isolates with MICs between 2 mg/L and 4 mg/L were identified in various regions [46,47,48], more frequent CET administration (600 mg every 8 h) was suggested to maintain serum concentrations higher than the MIC of the pathogen for a sufficient period during the interval between doses [49]. However, the superiority of this regimen has never been demonstrated. Similar variations have been suggested for the treatment of children [50]. For complicated pneumonia or other serious MRSA infections in children, some experts recommend administering ceftaroline over 2 h at a dose of 15 mg/kg (not to exceed 600 mg) every 8 h.

## 3. Glycopeptides

Glycopeptide antibiotics are actinomycete-derived drugs composed of glycosylated cyclic or polycyclic nonribosomal peptides that are effective against Gram-positive bacteria [7]. This antimicrobial activity is mainly due to the inhibition of bacterial cell wall peptidoglycan synthesis. Moreover, glycopeptides inhibit bacterial cell membrane permeability and affect bacterial RNA synthesis. Several drugs of this group, such as VAN, teicoplanin, telavancin, ramoplanin, and decaplanin, have been developed and studied for use in clinical practice [51]. However, only teicoplanin and, above all, VAN have been widely successful and continue to be prescribed. They remain the drugs of choice for the treatment of proven or suspected serious MRSA infections, although concerns regarding renal toxicity, emerging resistance, and administration challenges, including the lack of systemic absorption of the oral formulation, have driven research towards new antibacterial agents of these groups and led to the development of lipoglycopeptides [52,53,54,55,56]. These are antibiotics directly derived from VAN and teicoplanin but with improved antibacterial activity and, at least in some cases, more favourable pharmacokinetic properties. Among them, telavancin (TE) and oritavancin (ORI) have chemical structures quite similar to VAN but possess an additional lipophilic side chain attached to the disaccharide moiety and some other minor molecular modifications. These confer a different and more effective inhibition of bacterial cell wall peptidoglycan synthesis and a rapidly bactericidal character [57]. In contrast, dalbavancin (DA) is similar to teicoplanin, which already possesses a lipophilic chain but differs from this drug in several other features, including different characteristics and lengths of the sidechain. This allows an improved pharmacokinetic profile, although the mode of action is not substantially different from that of the parent molecule [58]. In vitro studies have shown that all of these drugs are significantly more effective than VAN against both MSSA and MRSA, and that most VAN-resistant *S. aureus* (VRSA) strains have a very low MIC. The FDA and EMA have licenced ORI and DA for the treatment of adult patients with complicated aSSTIs caused by susceptible isolates of Gram-positive bacteria, including both MSSA and MRSA [59,60]. TE is licenced for use in adults with aSSTIs and HABP/VABP [61]; however, it has been withdrawn from the market in Europe after a previous authorization [62]. Moreover, DA has been recently licenced for use in children from birth [63]. All are given intravenously, but due to their different pharmacokinetic characteristics, the schedule of administration of lipoglycopeptides differs significantly. In healthy adults, TE has a relatively short half-life (6.5 ± 0.9 h) and rapid total clearance (1.19 L/h) [64]. In contrast, DA and ORI have very long half-lives (approximately 2 weeks) with high protein-binding affinity (>90%) [65,66]. These differences explain why DA and ORI are given in a two-dose regimen, with each dose separated by one week or with a single higher dose, whereas TE is administered every 24 h for 7 to 14 days for SSSIs and for 7 to 21 days for HABP/VABP [66]. Several studies [67,68,69,70,71,72,73,74,75,76,77,78,79,80] have evaluated all these antibiotics in patients with complicated aSSTIs, generally evidencing that they were not inferior to traditional alternatives, including VAN, tedizolid, linezolid, and daptomycin. The safety and tolerability of DA and ORI are generally good. Adverse events are generally mild and transient and include injection site reactions, flushing, urticaria, pruritus, and nausea/vomiting. However, to minimize the risk of infusion-related reactions, ORI should be administered over 3 h, whereas DA can be given in approximately 30 min with some benefit for patients. Interestingly, in contrast to those receiving VAN, patients with a mild to moderate reduction in renal function receiving DA or ORI do not need blood concentration monitoring or drug dosage modification [67].

A meta-analysis of DA use in aSSTIs, including cases treated with two doses or with a single dose, revealed that, compared with traditional treatment, the clinical efficacy of this antibiotic was quite similar, regardless of the schedule used (two doses ORI, odds ratio [OR] 1.13; 95% CI 0.75–1.71; *p* = 0.55 or single dose ORI, OR 0.98; 95% CI 0.19–5.17; *p* = 0.98) [68]. However, microbiological assessment results indicated a favourable outcome for two doses compared to the single dose (OR 2.96; 95% CI 1.19–7.39; *p* = 0.02) in both MSSA and MRSA cases. The efficacy of DA in patients with Gram-positive infections, including *S. aureus*, was confirmed by a recent meta-analysis in which, together with studies enrolling aSSTI patients, patients with catheter-related bloodstream infections (CRBSIs) and osteomyelitis were included. In this study, the superiority of DA in comparison to standard treatment for the CRBSIs and osteomyelitis subgroups was evidenced [69].

The approval of DA for use in children was based on some pharmacokinetic studies [70,71] and a multicentre, open-label, actively controlled clinical trial enrolling paediatric patients from birth to less than 18 years of age with SSTIs [72]. Pharmacokinetic studies have reported that, to achieve drug exposure similar to that found effective in adults (1500 mg in single dose), doses of 18 mg/kg in older children and 22.5 mg/kg in neonates and children aged < 3 months were needed [70,71]. In the clinical trial, both single-dose and two-dose schedules were evaluated [72]. VAN for MRSA infections and oxacillin or flucloxacillin for MSSA infections were used as comparators. Early clinical response at 48 to 72 h (a ≥ 20% reduction in lesion size and no administration of rescue antibacterial therapy) was achieved in 97.3% of children receiving a single dose, in 93.6% of children in the two-dose group, and in 86.7% of children in the comparator arm [68].

ORI is licenced by the FDA [73] and EMA [74] for the treatment of aSSTIs in adults. Studies in patients with these diseases have shown the noninferiority of this drug compared with VAN [75]. The simplification of therapy with DA and ORI makes these drugs the best solution for the treatment of SSTIs in the ambulatory setting and emergency room provided that the patient can be carefully followed up at home. Moreover, compared with antibiotic alternatives such as vancomycin, DA and ORI allow significant economic advantages, mainly due to the reduction in the treatment duration [76].

In adults with aSSTIs, TE was found to be slightly more effective than VAN when MRSA was the infecting pathogen [77]. Among a group of 1500 patients with aSSTI, the clinical cure rate was 88.3% for patients given TE and 87.1% for those receiving VAN. However, in the case of MRSA, 90.6% of patients treated with TE and 84.4% of those treated with VAN were cured (95% CI for the difference, −1.1% to 9.3%) [77]. The efficacy of TE for the treatment of HABP was initially assessed with two identical, double-blind, controlled trials comparing this drug with VAN [78]. An analysis of the pooled clinically evaluable patients showed similar cure rates, with values of 82.4% for TE and 80.7% for VAN (95% CI for the difference, −4.3% to 7.7%). Similar results were obtained when only patients with *S. aureus* were isolated at baseline. The cure rates were similar for TLV and VAN (78.1% and 75.2%, respectively), including MRSA (74.8% and 74.7%, respectively) subsets. However, the cure rate among patients with MRSA with reduced susceptibility to VAN (MIC ≥ 1 µg/mL) was 87% in those treated with TE compared to 74% in those given VAN (95% CI 0.5–23.0) [78]. More recent studies have confirmed that TE is generally noninferior to VAN for the treatment of nosocomial pneumonia, with greater efficacy when MRSA is the cause of disease [79,80].

## 4. Oxazolidinones

Oxazolidinones are a recent class of synthetic antibiotics with a chemical structure characterized by a basic nucleus of 2-oxazolidone [81]. Oxazolidinones are effective orally or intravenously against multidrug-resistant Gram-positive bacteria, including MRSA and VAN-resistant *Enterococcus*. Moreover, most *Mycobacterium tuberculosis* strains are sensitive to oxazolidinones [77]. Antibacterial activity depends on the inhibition of protein synthesis through binding to the bacterial 23S ribosomal RNA of the 50S subunit. This prevents the formation of a functional 70S initiation complex, which is essential to bacterial RNA translation. As no other antibiotic possesses this mechanism of action, no cross-resistance between oxazolidinones and other protein-synthesis inhibitors can occur [82].

The first licenced oxazolidinone was linezolid, which was found to be effective in several clinical trials enrolling patients with aSSTIs, CAP, nosocomial pneumonia, and tuberculosis [83,84,85]. However, emerging linezolid resistance has been repeatedly reported with increased difficulties in the treatment of certain infectious diseases [86]. Moreover, linezolid pharmacokinetic characteristics and safety profiles are not ideal, especially for children. The pharmacokinetics of the drug can significantly vary from subject to subject according to body weight, age, and renal and hepatic function, and co-medications are the most important factors that are indications for drug-level monitoring and dosage adjustment. Unlike in adults, for whom this drug can be administered every 12 h, in children, linezolid must be given three times daily [87,88,89,90,91,92]. Furthermore, toxicity associated with prolonged use, mainly myelosuppression but also lactic acidosis and peripheral and ocular neuropathies, has been repeatedly reported in both adults and children [93,94,95] Finally, linezolid has a chemical structure quite similar to the reversible MAO inhibitor toloxatone and is a weak, reversible inhibitor of MAO-A and MAO-B isoforms. This may lead to peripheral or central neurotransmitter accumulation, with potentially serious consequences. When taken in combination with vasoconstrictors, such as pseudoephedrine, or high dietary tyramine, it can cause sudden blood pressure elevations that may lead to hypertensive crises. Combination with serotonergic agents may lead to rare, but potentially life-threatening, serotonin syndrome [96,97]. Tedizolid (TD) is the second oxazolidinone antibiotic that has been licenced by the FDA [98] and EMA [99] for use in adults to treat aSSTIs caused by designated susceptible bacteria. Similar to linezolid, it can be given by mouth or intravenously. However, it is active in vitro against almost all MRSA isolates, including several of those resistant to linezolid. A meta-analysis of the studies published up until December 2017, evaluating the in vitro activity of TD against 10,119 MRSA strains, showed a pooled prevalence of susceptibility of 99.6% (95% CI 99.5–99.8) [100]. The efficacy against linezolid-resistant strains was 100% in one study and slightly lower than 50% in three other studies. The MIC90 of TD against MRSA varied between 0.25 mg/L and 0.5 mg/L, whereas that against linezolid was 2 mg/L [101,102,103,104]. TD has more favourable pharmacokinetic properties that allow for once-daily dosing in both adults and children older than 2 years of age [99]. Moreover, TD has better tolerability and safety. Compared to linezolid, TD administration is associated with a lower incidence of nausea (OR 0.68, 95% CI 0.49–0.94) and vomiting (OR 0.56, 95% CI 0.34–0.96), a lower risk of bone marrow suppression (1.3% vs. 3.9%; OR 0.36, 95% CI 0.17–0.76), and a lower risk of thrombocytopenia, although this is not significant (4.2% vs. 6.8%; OR 0.61, 95% CI 0.25–1.49) [105]. However, contrarily to linezolid, provocative testing in humans and animal models has failed to uncover significant signals that would suggest a potential for hypertensive or serotonergic adverse consequences at the therapeutic dose of TD [106].

From a clinical point of view, the impact of TD on SSTIs has been clearly defined by a recently published meta-analysis of four studies that showed that, in adults, TD was noninferior to linezolid [107]. A total of 2056 adult patients were enrolled. The early clinical response rates were 79.6% and 80.5% for patients receiving TD and linezolid, respectively. The pooled analysis showed that TD had a noninferior early clinical response rate compared with linezolid (OR 0.96, 95% CI 0.77–1.19, I2 = 0%) regardless of the type of aSSTI (cellulitis/erysipelas: 75.1% vs. 77.1%; OR 0.90, 95% CI 0.64–1.27, I2 = 25%; major cutaneous abscess: 85.1% vs. 86.8%; OR 0.93, 95% CI 0.42–2.03, I2 = 37%; and wound infection: 85.9% vs. 82.6%; OR 1.29, 95% CI 0.66–2.51, I2 = 45%). For MRSA patients, the microbiological response to TD (95.2%) was comparable to that to linezolid (94%) (OR 1.19, 95% CI 0.49–2.90) [107].

## 5. Tetracyclines

Tetracyclines are an older group of antimicrobials that were largely used in the first years of the antibiotic era but were progressively abandoned because of the emergence of resistance in most of the pathogens that were initially sensitive [108]. Recently, novel tetracyclines able to overcome common tetracycline resistance mechanisms, such as efflux and ribosomal modifications, have been developed. The first of these novel tetracyclines was tigecycline, a drug that was found to be effective against most Gram-positive bacteria, including MRSA, several important Gram-negative rods, and atypical bacteria [109]. However, tigecycline has some limitations that have discouraged its widespread use. Tigecycline has very low bioavailability and must only be used intravenously. Moreover, its safety and tolerability are debated, as patients receiving this drug have been found to be at increased risk of mortality, and frequently suffer from nausea and vomiting that is sometimes severe enough to require drug discontinuation [109].

The possibility of overcoming tigecycline limitations without a reduction in microbial efficacy explains the interest shown by physicians in a more recent new tetracycline, omadacycline (OM) [110]. OM remains highly effective against Gram-positive bacteria, including MRSA, penicillin-resistant and multidrug-resistant *Streptococcus pneumoniae*, and VAN-resistant *Enterococcus* spp. OM is also active against pathogens that are important in community-acquired respiratory tract infections, including *Haemophilus influenzae* and *Moraxella catarrhalis* [110]. Moreover, OM has a 34.5% bioavailability in healthy adult subjects that allows its oral administration. Furthermore, it has a very long half-life (17 h) that allows single daily administration and it seems to be significantly better tolerated than tigecycline, as nausea and vomiting in patients receiving therapeutic doses have been reported to be less common and less severe [111]. From the available studies, the FDA has licenced OM for the treatment of aSSTIs and CAP [112]; however, the drug is not licenced in Europe [113]. Both aSSTIs and CAP can be treated with initial intravenous administration (a loading dose of 200 mg IV once or 100 mg IV twice on Day 1) followed by 100 mg IV or 300 mg orally daily for 7–14 days. For aSSTIs only, OM can be given orally at the initiation of treatment (450 mg on Days 1 and 2, followed by 300 mg orally daily for 7–14 days). No dose adjustments are required according to age, sex, or liver or kidney function. The US licence was based on two aSSTI studies and one CAP study. In both aSSTI studies, which were randomized, double-blind, double-dummy studies, OM was compared to linezolid. In the first study [111], the two drugs were initially given intravenously with the option to transition to an oral preparation after ≥3 days. In the second study [112], only oral doses were given. In both studies, OM was not inferior to linezolid in terms of either early or post-treatment response, regardless of the type of aSSTI and baseline pathogen, including cases due to MRSA [114,115]. In a pooled analysis, early clinical response, defined as patient survival with a reduction in the lesion area of at least 20% after 48–72 h, was shown in 86.2% and 83.9% (95% CI for the difference −1) of patients receiving OM or linezolid, respectively. Evaluation revealed that success, defined as the resolution of infection without the need for further antibiotic administration 7–14 days after the last treatment dose, was achieved in 85.1% and 82.1% (difference 2.9; 95% CI −1.0 to 6.9) of patients receiving OM or linezolid, respectively. Adverse events occurred with similar frequencies (51.1% and 41.2% in OM and linezolid patients, respectively). Although nausea and vomiting were the most common adverse events in these studies, they were not severe enough to lead to drug discontinuation [114,115].

In CAP, OM has been found to be noninferior to moxifloxacin. Early clinical response, defined as symptom improvement 72–120 h after the first dose of the drug, no use of rescue antibiotics, and patient survival, was achieved in 81.1% vs. 82.7% of patients (difference −1.6; 95% CI −7.1 to 3.8) [116]. Similar results were obtained when post-treatment efficacy was evaluated (87.6% vs. 85.1%; difference, 2.5; 95% CI −2.4 to 7.4). In this study, tolerability was also good, with only a few patients suffering from diarrhoea. No *Clostridium-difficile*-associated diarrhoea was reported [116].

No study has been carried out in children. However, as OM shares the tetracycline-class effects of tooth discoloration, the inhibition of bone growth, and a potential effect on anticoagulants [117], it seems highly likely that this drug will not be evaluated in children, particularly in those younger than 8 years of age, in whom tetracycline use is not currently recommended [118].

## 6. Quinolones

Generally, quinolones, including fluoroquinolones, have poor activity against *S. aureus*, particularly MRSA. A study their testing activity against 107 MRSA strains showed that ciprofloxacin, ofloxacin, gatifloxacin, and levofloxacin were ineffective against these pathogens in 92.5%, 80.4%, 53.3%, and 49.5% of cases, respectively [119]. Moreover, with use, resistance to other previously sensitive bacteria has emerged. To overcome these problems, attempts to develop new quinolones with improved antibacterial activity have been made.

The first new quinolone that was able to overcome old and emerging bacterial resistance among quinolones was delafloxacin (DL). Significant modifications to the quinolone structure have been performed, and this has led to the synthesis of a molecule that conserves the activity against Gram-negative rods of fluoroquinolones. Moreover, this has resulted in acquired activity against most Gram-positive bacteria, including more than 99% MSSA and 91.2–95.3% MRSA [120]. The drug, which has been prepared for both oral and intravenous administration, is presently licenced in the USA for the treatment of aSSTIs and CAP [121], and in Europe only for aSSTIs [122]. DL has good bioavailability (approximately 60%), is approximately 80% bound to plasma proteins, and has a mean half-life of approximately 4 h. This explains the suggested dosages for both aSSTIs and CAP of 300 mg intravenously every 12 h or 450 mg orally every 12 h for 5 days to 10 days for CAP, and to 14 days for ASSTIs [121]. The efficacy of DL in aSSTIs has been demonstrated in two large randomized, double-blind, double-dummy, multinational, phase 3 noninferiority trials [123,124]. Cellulitis, wound infection, major cutaneous abscess, and burn infections were the most commonly treated aSSTIs in both trials, with rates of 39%, 35%, 25%, and < 1% in the first study [123] and 48%, 26%, 25%, and 1% in the second study [12], respectively. DL was compared with the combination of vancomycin plus aztreonam in patients with similar baseline characteristics in terms of the type of aSSTI, age, sex, and underlying conditions. In both studies, the results showed the noninferiority of DL compared with the vancomycin plus aztreonam combination; *S. aureus* eradication was achieved in more than 98% of cases, regardless of *S. aureus* susceptibility to methicillin [123,124]. Interestingly, microbiological evaluation showed that the MIC for DL was very low (0.25 µg/mL), whereas all of the other tested quinolones were microbiologically ineffective. The use of DL in adults with CAP has confirmed the expected efficacy suggested by microbiological evaluations. Microbiological success rates were higher than 90% for all aetiological agents, and values of 100% were reached in a few cases due to MRSA [125]. No study has been performed in children. Although quinolones have been authorized for use in selected paediatric populations when other drugs that are effective against the supposed or demonstrated infecting pathogen(s) are not available, the risk that children may develop severe musculoskeletal disorders when treated with quinolones remains a relevant limitation to the execution of paediatric trials with these antibiotics [126].

## 7. Conclusions

This paper reported the main characteristics of the most recently authorized drugs for treatment of some of the most common *S. aureus* infections (Table 1). Compared to previously reported studies concerning the same topic, this paper includes the most recent studies and offers the reader a more complete and reasoned therapeutic choice.

The main reasons for the development of new anti-*S aureus* drugs were the intent to overcome the emerging resistance of *S. aureus* to the drugs currently prescribed against this pathogen and to reduce the risk of adverse events frequently associated with traditional therapy [127,128]. Drugs belonging to five antibiotic classes have been developed and those presently authorized for use by the FDA and/or EMA have been discussed. The results are encouraging because in vitro studies have shown that these new drugs have better antimicrobial activity and, at least in some cases, more favourable pharmacokinetic properties, in addition to higher safety and tolerability compared with the presently available anti-staphylococcal drugs. This indicates their potential use in reducing the risk of failure of *S. aureus* therapy. However, an in-depth analysis of microbiological and clinical studies carried out with these new drugs seems to indicate that the conclusions drawn from the available data may lead to evaluations that are slightly too optimistic. Moreover, many studies still need to be conducted before the problem of resistance of *S. aureus* to the antibiotics available today can be completely solved. Several new drugs have a significantly broader spectrum of activity than those presently used to treat *S. aureus* infections [23,129]. This means that their use may favour the emergence of resistance of the relevant bacteria involved in the determination of severe infections, reducing the efficacy of these drugs in the emerging therapy of severe infections of undetermined origin. Moreover, despite having better antimicrobial activity, most clinical studies simply indicate that these new drugs are noninferior to traditional antibiotics. The superiority of new antibiotics in comparative studies including a relevant number of patients has not been demonstrated. Furthermore, most, if not all, of the clinical trials that have led to the approval of these new drugs by the FDA and EMA have been carried out in patients with aSSTIs or different types of pneumonia. Very few patients with other types of *S. aureus* infections, such as bacteraemia and osteomyelitis, have been included in clinical trials. Finally, regarding the use of most of these new drugs in children, a topic that has only been marginally considered in previous reviews [130], very few trials have been performed. In some cases, pharmacokinetic and clinical studies to decide the best dosages of each drug for children of different ages with different *S. aureus* infections have not been conducted. In other cases, such as for drugs included in the tetracycline and quinolone groups, use in children is limited owing to the risk of adverse events.

Considering the overall available research, the new anti-*S. aureus* drugs appear to present a great therapeutic opportunity for overcoming resistance to traditional therapy with advantages in the pharmacokinetic characteristics of some of these drugs and a potential reduction in hospital stays and economic costs derived from their use.

## Figures and Tables

**Table 1:** Main approved new oral and intravenous drugs for the treatment of *Staphylococcus aureus* infection.

Drug Class	Cephalosporins	Lipopeptides	Lipoglycopeptides	Oxazolidinones		Tetracyclines	Fluoroquinolones	
Drug Name	Ceftobiprole	Ceftaroline	Telavancin	Dalbavancin	Oritavancin	Tedizolid	Omadacycline	Delafloxacin
**In vitro activity**	MSSA, MRSA, CoNS, *streptococci,* penicillin-R *S. pneumoniae* and *E. faecalis* Gram-negative pathogens including *Pseudomonas aeruginosa*	MSSA, MRSA, hVISA, VISA, VRSA and DAP-non susceptible *S. aureus,* CoNS, *streptococci* penicillin-R *S. pneumoniae* Gram-negative pathogens excluding *Pseudomonas aeruginosa*	MSSA, MRSA, CoNS, streptococci, enterococci including VRE *vanB*	MSSA, MRSA, CoNS, streptococci, enterococci including VRE *vanB*	MSSA, MRSA, CoNS, streptococci, enterococci including VRE *vanA, vanB*	MSSA, MRSA, CoNS, streptococci, enterococci including VRE *vanA, vanB*	MSSA, MRSA, CoNS, streptococci, *E. faecFalis*	MSSA, MRSA, CoNS, streptococci, enterococci including VRE *vanA, vanB* Stable in the presence of ESBLs
**Drug target**	Cell wall synthesis	Cell wall synthesis	Cell wall synthesis	Cell wall synthesis	Cell wall synthesis	Protein synthesis	Protein synthesis	DNA replication
**Type of activity**	Bactericidal	Bactericidal	Bactericidal	Bactericidal	Bactericidal	Bacteriostatic	Bacteriostatic	Bactericidal
**Half-life (h)** **-** **Oral** **bioavailability** **(%)**	2–3	2–3	8	192–336	393	10 - 91	17–21 - 34.5	8 - 58.8
**Doses, frequency and duration**	IV: 500 mg over 2 h t.i.d.	IV: 600 mg over 60 min b.i.d./t.i.d. in severe infections	IV: 10 mg/kg q.d.	IV single-dose regimen 1500 mg over 30 min For sequential use: 1500 mg on day 1 and 1000/ 1500 mg every 2 weeks	IV single-dose regimen: 1200 mg over 3 h For sequential use: 1200 day 1 and then 800/1200 mg once week	Oral: 200 mg IV: 200 mg over 1 h q.d.	Oral: loading dose 450 mg, then 300 mg IV: loading dose 200 mg, then 100 mg over 30 min q.d.	Oral: 450 mg IV: 300 mg over 1 h b.i.d.
**Protein Binding (%)** **Excretion**	16 Faeces: 6% Urine: 88%	20 Faeces: 6% Urine: 88%	90 Faeces: <1% Urine: <76%	93–98 Faeces: 20% Urine: 45%	85 Not metabolized	70–90 If oral: Faeces: 82% Urine: 18%	20 If oral: Faeces: N/A Urine: 27% If IV: Faeces: 81% Urine: 15%	84 If oral: Faeces: 28% Urine: 65% If IV: Faeces: 48% Urine: 50%
**Doses adjustments not required for**	CrCL > 50 mL/min	CrCL > 50 mL/min	CrCL > 50 mL/min	CrCL > 30 mL/min	Renal impairment, hepatic impairment	Hepatic dysfunction, renal dysfunction	Hepatic impairment, renal impairment	Body weight, hepatic impairment, mild-to-moderate renal impairment
**FDA or EMA approval** **(Year and indications)**	Not approved by the FDA 2009 ABSSSI, CAP, HAP	2010 ABSSSI, CAP	2009, ABSSSI, HAP, VAP	2014, ABSSSI	2014, ABSSSI	2014, ABSSSI	2018 ABSSSI, CAP	2017 and 2019, ABSSSI, CAP
**Paediatrics Therapeutic indication**	No data	Yes	No data	Yes	No data	Yes >12 years	Not approved	Not approved
**Future directions and points of clinical interest**	VAP	Primary SAB, complicated SAB secondary to non-ABSSSI causes (IE, OSM, or non-responsive to first line therapy)		OSM; prosthetic infection including IE, CLABSI, OPAT regimens	OSM; prosthetic infection including IE, CLABSI, OPAT regimens	OSM; HAP, or VAP due to MRSA Especially if resistant or intolerant to linezolid	HAP, biliary infections and OSM to allow early hospital discharge	HAP, MRSA OSM

Abbreviations: MSSA, methicillin-sensitive *Staphylococcus aureus;* MRSA, methicillin-resistant *S. aureus;* CoNS, coagulase-negative staphylococci; VRE, vancomycin-resistant *E. faecium;* ABSSSI, acute bacterial skin and skin structure infections; BSI, bloodstream infections; SAB, *S. aureus* bacteraemia; IE, infective endocarditis; CAP, community-acquired pneumonia; HAP, hospital-acquired pneumonia; VAP, ventilator-assisted pneumonia; CLABSI: catheter-related bloodstream infection. IV, intravenous; PO, per os; OSM; osteomyelitis; PJI, prosthetic joint infection; q.d., quaque die/once daily; b.i.d., bis in die/twice daily; t.i.d., ter in die/three times daily; CPK, creatinine phosphokinase; FDA, Food and Drug Administration; EMA, European Medicines Agency; SA, *S. aureus*; OPAT: outpatient parenteral antimicrobial therapy.

## Data Availability

Not applicable.
